# Mechanosensing in Myosin Filament Solves a 60 Years Old Conflict in Skeletal Muscle Modeling between High Power Output and Slow Rise in Tension

**DOI:** 10.3389/fphys.2016.00427

**Published:** 2016-09-23

**Authors:** Lorenzo Marcucci, Carlo Reggiani

**Affiliations:** ^1^Department of Biomedical Sciences, University of PaduaPadua, Italy; ^2^Centre for Mechanics of Biological Materials, University of PaduaPadua, Italy

**Keywords:** muscle, modeling, super-relaxed state, myosin motors, power output

## Abstract

Almost 60 years ago Andrew Huxley with his seminal paper (Huxley, 1957) laid the foundation of modern muscle modeling, linking chemical to mechanical events. He described mechanics and energetics of muscle contraction through the cyclical attachment and detachment of myosin motors to the actin filament with *ad-hoc* assumptions on the dependence of the rate constants on the strain of the myosin motors. That relatively simple hypothesis is still present in recent models, even though with several modifications to adapt the model to the different experimental constraints which became subsequently available. However, already in that paper, one controversial aspect of the model became clear. Relatively high attachment and detachment rates of myosin to the actin filament were needed to simulate the high power output at intermediate velocity of shortening. However, these rates were incompatible with the relatively slow rise in tension upon activation, despite the rise should be generated by the same rate functions. This discrepancy has not been fully solved till today, despite several hypotheses have been forwarded to reconcile the two aspects. Here, using a conventional muscle model, we show that the recently revealed mechanosensing mechanism of recruitment of myosin motors (Linari et al., 2015) can solve this long standing problem without any further *ad-hoc* hypotheses.

## Introduction

Muscle contraction is generated by the cyclical interaction of myosin motors with the actin filament. Following attachment the myosin motor produces force and relative filaments sliding. This cyclical interaction has been extensively studied and characterized leading to the definition of the myosin-actin cycle. The kinetics of the transitions between the myosin states in the cycle are responsible for both the isometric contraction, when, following activation, the tension rises to a maximum steady value, and the isotonic contraction, when the muscle shortens at a constant velocity which increases with the reduction of the load, according to the force-velocity relation (Hill, 1938). Muscle models based on relatively simple myosin-actin cycles, show that the kinetics needed to simulate the high-power output at intermediate shortening velocities, imply a rate of tension development in isometric contraction much faster than that experimentally observed (Piazzesi and Lombardi, 1995; Huxley and Tideswell, 1997; Månsson, 2010; Marcucci and Yanagida, 2012; Caremani et al., 2013). Despite decades of experimental discoveries and characterization of a more rich environment in the cross bridge cycle, apparently no simple explanation is able to reconcile this controversial behavior. Different additional mechanisms have been introduced on the myosin-actin cycle to solve the problem: a non-conventional path in cross-bridge cycle (Piazzesi and Lombardi, 1995), an active role of the second myosin head (Huxley and Tideswell, 1997), a dependence of the rate of attachment on the shortening velocity (Månsson, 2010), the possibility that one (Caremani et al., 2013) or more actin monomers become available for the same attached myosin during one ATPase cycle (Marcucci and Yanagida, 2012). None of these hypotheses, however, provided a definite convincing solution of the problem.

A recent breakthrough on thick filament structure has substantiated the existence of two distinct configurations of the myosin motors in the detached state. In one configuration, motors at rest lie along the thick filament backbone, regularly packed in quasi-helical grooves, as originally demonstrated with cryo-electromicroscopy (Woodhead et al., 2005), while in the second configuration they rise toward the thin filament. The presence in relaxed skinned fibers of two populations of myosin motors, one with a low ATP hydrolysis rate (super-relaxed or OFF state) and the other, more disordered (active or ON state) with ten times higher ATPase rate, has been first shown by Cooke in relaxed skinned fibers (Stewart et al., 2010). The muscle activation leads to a transition of the myosin motors from the OFF state to the ON state. Only in this latter state, myosins can bind the actin filaments and perform the power stroke that leads to generation of force and/or filament displacement. Recently published evidence on intact frog muscle fibers at low temperature, shows that the rise of filament stress accompanying high load contraction induces progressive recruitment of motors for the interaction with the actin (Linari et al., 2015). At zero or very low tensions (less than one tenth of the maximum isometric tension *T*_0_), only a very few myosin motors are in the ON state, i.e., ready to bind actin filament, and are responsible for the rapid development of the ability to shorten at the maximum velocity (Lombardi and Menchetti, 1984). The number of motors in the ON state increases during the rise of isometric tension and reaches the maximum value at about 0.5 *T*_0_. In support to the stress dependent transition, keeping to zero the tension of a contracting muscle fiber with the imposition of shortening at the maximum velocity *V*_0_ for 20–40 ms, sensibly reduces the population in the active state, favoring the transition from the ON to the OFF state. The change in the proportion between the two populations, affects the time required for the recovery of a steady tension when the isometric condition is restored. The time constant for the rise in tension after initial activation, thus starting from almost all myosins in the OFF state, has been estimated at about τ_*R*_ = 34 ms. After an unloaded period of 40 ms, corresponding to a shortening of 10% of the muscle length, the tension recovery is faster, with a time constant of τ_10_ = 28 ms, because only a part of the ON myosins turns OFF. After an unloaded period of 20 ms, corresponding to a shortening of 5% of the muscle length, the time constant is even lower, τ_5_ = 24 ms, because an even minor part of myosins became OFF (Linari et al., 2015). In our view, this imply that the characteristic time scale of the ON-OFF transition is, then, in the same order of magnitude of the attachment detachment process. Consequently, this regulatory mechanism may be responsible for the relatively slower tension rise following activation compared to the relatively faster attachment and detachment rates required for the high power output at intermediate velocities of shortening.

In the present work we test the above hypothesis introducing the experimentally observed mechanosensing mechanism into a conventional model recently designed by one of the authors (Marcucci et al., 2016). At the best of our knowledge, this is the first time that such a mechanism is embedded in the cross-bridge cycle governing a muscle contraction model. The conventional model is able to reproduce a number of experimental data, but it shares the common limit of mathematical models described before: the maximum power output is relatively low compared to the experimental data, because of a relatively high inflection in the tension-velocity curve (Figure 4 in Marcucci et al., 2016). In our conventional model, this limit is a consequence of the relatively slow attachment rate of fresh myosin motors, imposed to achieve a physiological time course of tension development upon activation, as also observed in Månsson (2010). The same reason is probably true in other models, even though it is not explicitly said. Starting from that conventional model, we imposed the observed thick filament tension dependence on the rate constants between the two non-force generating states, here described in term of OFF state and ON state. This modification introduces the mechanosensing mechanism. The description of all other stable states are kept the same. We then compare the simulated behavior to highlight the physiological meaning of the observed property.

## Materials and methods

**Figure 1:**
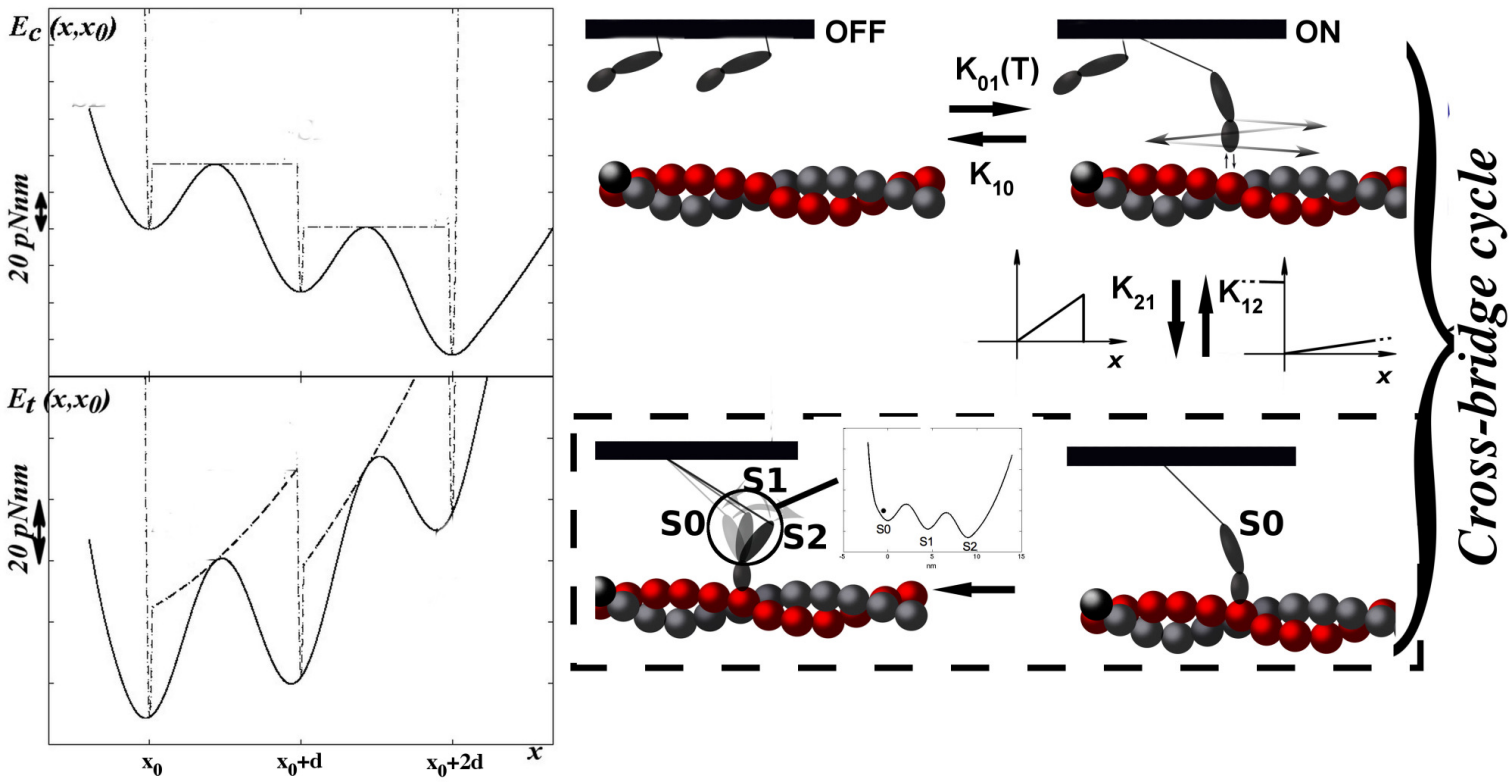
**MS model**. The model is based on a conventional model (Marcucci et al., 2016) with the inclusion of the mechanosensing mechanism in the rate constants between the two non-force generating states. The conventional model differs only for the absence of the mechanosensing mechanism and the inclusion of the classic actin-based cooperativity (see Materials and Methods and Supplemental Information). Rates for the attachment to and detachment from strongly attached states are functions of the strain in the *i*−*th* motor (*x*_*i*_), following the original dependence hypothesized by Huxley (1957). The power stroke has two steps as reported in the left top panel. Actomyosin energy is biased by $8K\bar{T}$ every step. Left bottom figure represents the actomyosin energy plus the elastic component due to the myosin stiffness. *x*_0_ is the extension at the time of attachment, zero in figure.

In this paper we test the above mentioned hypothesis comparing the two mathematical models using a Monte-Carlo numerical method. We will refer to the original model as conventional model and to the new one as mechanosensing (MS) model. The conventional model is described in Marcucci et al. (2016), we resume its main features here and describe the modifications to include the mechanosensing mechanism. The model (Figure 1) describes a single half-sarcomere with *N*_*fil*_ couples of thin and thick filaments and *N*_*xb*_ myosin motors per thick filament (compatible with the physiological values in the 2-D simplification, see Table S1 and Supplemental Information). We simulate the tension-time and tension-velocity curves of the whole fiber, under the hypothesis of homogeneous behavior in the series of sarcomeres. Importantly, the experimental reference is given by the frog muscle fiber contractile behavior at 4°C as described in Piazzesi et al. (2007), Linari et al. (2015), and Lombardi et al. (1992).

Both filaments are rigid. Actin filaments are constrained to have the same value in the *z*-direction through the Z-line which represents the coupling term in the mathematical model. *z* = 0 refers to the optimal length of the sarcomere. Myosin motors are attached through an elastic element to the thick filament backbone and can cyclically interact with an actin filament. Their position is defined by *x*_*i*_ (i = 1, 2…*N*_*xb*_), the extension of the motor elastic element. Myosin motor has then five stable states in both models: two non-force-generating states and three force-generating states (including a two step power stroke). In MS model, the non-force-generating states correspond to the super-relaxed, of OFF state, with a negligible ATP hydrolysis, and to the disordered relaxed, or ON, state. In the conventional model, they are representative of the detached and weakly attached state. For simplicity we keep the OFF and ON nomenclature. Motors in the ON state are ready to attach to actin filament forming the strongly attached states. In the conventional model, the OFF and ON transition rates, *k*_01_ and *k*_10_, respectively, include the cooperativity between myosin motors. Cooperativity affects the transition rates *k*_01_ and *k*_10_ through a parameter γ^*n*^, where *n* is 0 if there are no nearest neighbors and 1 or 2 if one or two, respectively, myosin motors are already weakly or strongly attached. Then $k_{01}=k_{01}^{0}\gamma^{n}$ and $k_{10}=k_{10}^{0}\gamma^{-n}$ (Rice et al., 2003). Such cooperativity is related to the displacement of the tropomyosin filament generated by an attached myosin motor, thus it is related to the thin filament.

**Figure 2:**
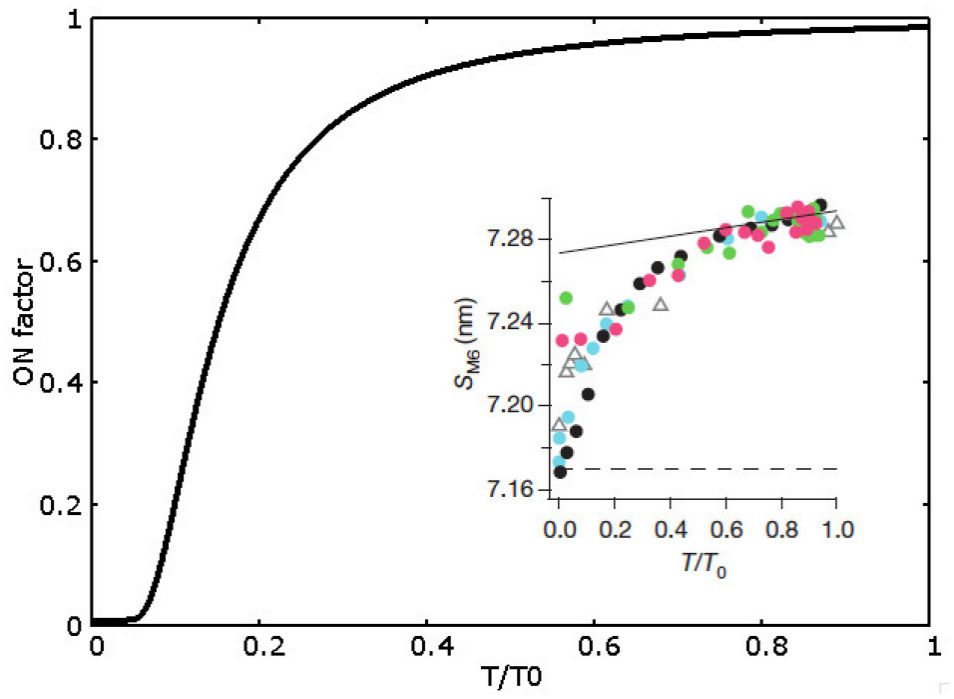
**ON factor dependence from the relative tension on the thick filament**. Experimental data (shown in the inset, adapted by permission from Macmillan Publishers Ltd: Nature Linari et al., 2015, copyright 2016) refers to SM6 signal and a direct comparison with the model prediction is not possible (see text). Therefore, ON factor has been defined to assure (i) about 5% of ON motors a low forces (less than 0.1 *T*_0_), and (ii) a saturating effect at forces higher than 0.5 *T*_0_, where almost all motors are in the ON state.

In MS model, we abandon the cooperativity mechanism, instead we introduce the novelty of the mechanosensing mechanism, i.e., a stress dependent regulation based on the thick filament. The OFF to ON transition rate depends on the tension born by the thick filament as observed experimentally (Linari et al., 2015). As described in the introduction, the probability of switching between ON and OFF depends on the tension in the thick filament. Experimentally, the mechanosensing mechanism has been studied through the X-ray diffraction method, in particular analyzing the spacing of M6 reflection at different tensions acting on the thick filament backbone. It is not straightforward to make a direct relationship between that observable and the myosins distribution in the model since it would require a detailed description of the three-dimensional geometry as well as some hypotheses on the backbone elasticity which may be not linear. Then, as the simplest hypothesis, the OFF to ON rate (*k*_01_) is a constant multiplied by a factor *ON*_*f*_(*T*), which is a function of the tension borne by the thick filament *T*. *ON*_*f*_(*T*) is defined as $ON_{f}\left(T\right)=m+\left(1-m\right)exp\left(-t_{f}/T^{2}\right)$. The shape of this function, shown in Figure 2, closely mimics the obtained experimental observations (values of the parameters are reported in Supplemental Information and Table S1).

The inverse transition rate, *k*_10_, has been chosen constant as the simplest hypothesis. The ratio of these two rates is chosen to allow for about 5% of myosin motors in the ON state when muscle is relaxed, as hypothesized in the experimental work.

In the ON state, myosins are in equilibrium with the strongly attached state. Both the conventional and MS model have three strongly attached states (S), in either pre-power stroke (S0), first (S1) or second (S2) post-power stroke. In the MS model, myosin motors detach from the strongly attached state switching to the ON state, while in the conventional model the detachment lead to a OFF state. In the OFF state, myosin motors are completely prevented to strongly attach to the actin filament.

From here on the models are equal. The attachment occurs only in the pre-power stroke state, while the detachment can occur in both the pre and the post power stroke state. The attachment to and detachment from the S state follow the classical H57 hypothesis (Huxley, 1957). The attachment rate *k*_12_ is non-zero only in the stretched configuration of the elastic element (*x* > 0), increases linearly with *x* up to *x*_*lim*_. The detachment rate, $k_{20}^{+}$ or $k_{21}^{+}$ for the conventional (S to OFF) or the MS (S to ON) model respectively, also increases linearly with *x* > 0, and attains a very high value when *x* < 0 ($k_{20}^{-}$ or $k_{21}^{-}$). The former hypothesis, related to the Brownian search and catch mechanism, has an original observations in Myosin V (Fujita et al., 2012), and has been extensively used in muscle modeling under different modifications. If the absolute value of *x* exceeds 20 *nm*, mechanical dislodging occurs. Numerical values used for the models are resumed in Table S1.

In both models Calcium concentration is saturating and thin filament reaches the full activation within the first millisecond (Fusi et al., 2014) (see Supplemental Information in Marcucci et al., 2016). The strongly attached state is described as a continuous energy landscape with three minima, corresponding to a pre-power stroke and a power stroke with two steps. The central region of the energy landscape is defined as:

(1) $$\begin{array}{l} E_{c}\left(x\right)=H\sin\left(2\pi x/d+\alpha_{d}\right)+F_{atp}x \end{array}$$

*x* is the extension of the elastic element in each myosin motor, respect its anchor on the thick filament. *H* is the energy barrier in the sinusoidal function, and is chosen to be $5.8K\bar{T}$. ATP energy release favors the power stroke by biasing the biochemical energy toward the post power stroke. To simulate this bias, we added a linear drop of $8K\bar{T}$ (*K* is the Boltzmann constant and $\bar{T}$ denotes the absolute temperature) every *d*, the distance between two consecutive minima, to the flat sinusoidal part, then $F_{atp}=8K\bar{T}/d$. α_*d*_ is a constant angle that adjusts for the constant ATP drop by shifting the first minimum to *x* = *x*_0_, the extension at the time of attachment. *x*_0_ depends on the H57 hypothesis plus a random component within [−2.5 nm, 2.5 nm] to simulate the actin diameter (Huxley and Tideswell, 1996). We can then approximate the minimum in the energy as a parabola with stiffness 48 *pN*/*nm*, much higher than the 2 *pN*/*nm* stiffness of the myosin motor. Meanwhile, the convex part is expressed by a polynomial, ensuring a continuous first derivative.

The lengths of the actin and myosin half-filaments are LA = 1224 nm, and LM = 825 nm, respectively. The myosin half-filament includes LB = 50 nm of bare zone with no myosin motors. In sarcomere, myosin molecules are spaced by 14.3 nm, implying about 54 myosin molecules per half-thick filament. Each thick filament is surrounded by six double-helix thin filaments, each of which can be reached by the myosin heads on three thick filaments; therefore, each myosin filament contains approximately 27 double-headed myosin molecules, which can interact with each actin filament. To allow for possible interaction from other myosin heads (Spudich, 2014), we increase the number of myosin heads interacting with each actin filament to 76, as in Marcucci et al. (2016).

## Results

**Figure 3:**
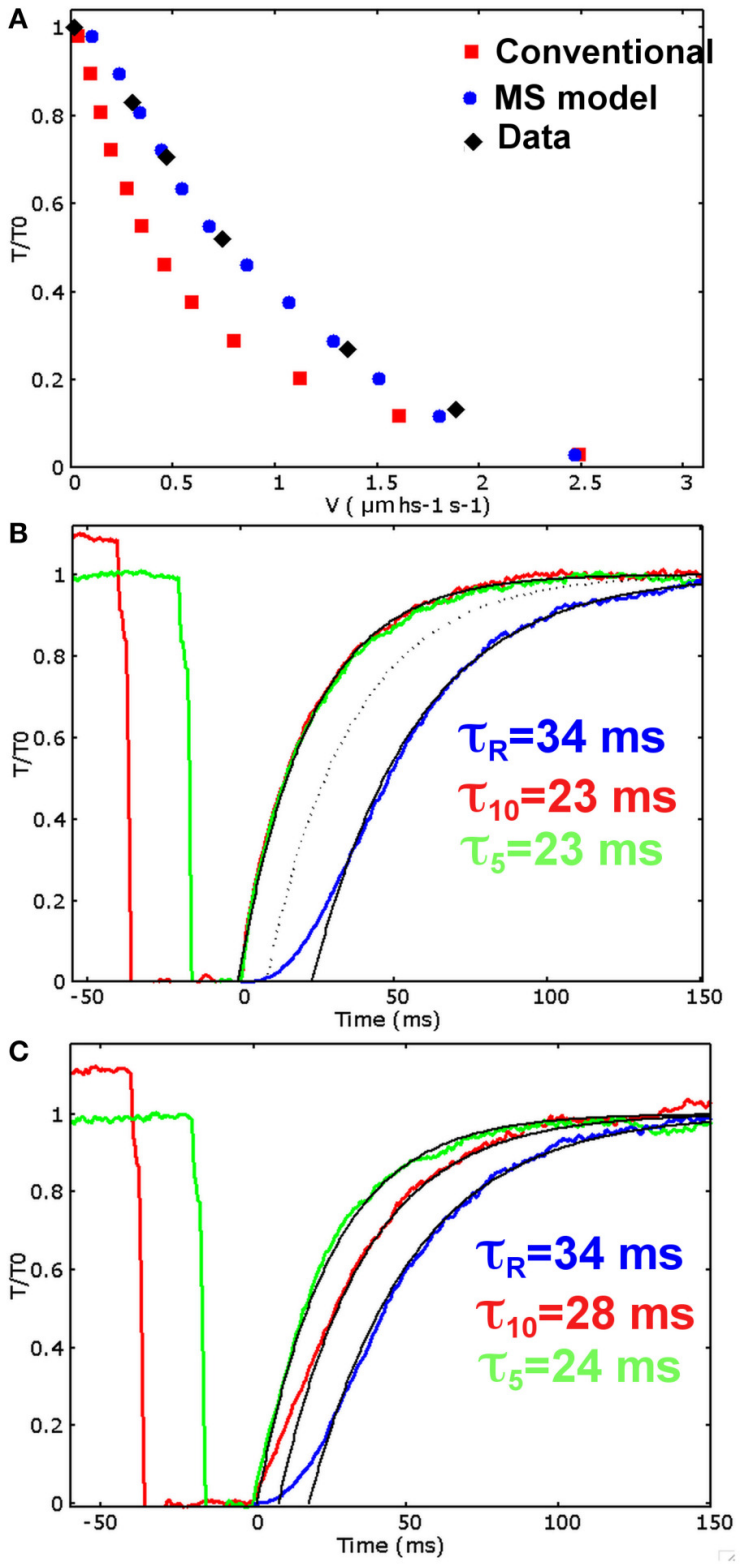
**Simulations for the two models. (A)** Simulated and experimental data for the tension-velocity curves. Experimental data: filled diamond (from Piazzesi et al., 2007), conventional model: red squares, MS model: blue dots. **(B)** Conventional model. Simulated rise in tension after initial activation (blue), and tension recovery after 40 (red) and 20 (green) milliseconds at zero tension shortening phase, corresponding to 10% and 5% of muscle length shortening respectively. The rise in tension after activation is comparable with the experimental data, as well as the recovery after 20 ms at zero tension, but the recovery rate does not depend on the duration of the zero tension period. **(C)** MS model. Simulated rise in tension after initial activation (blue), and tension recovery after 40 (red) and 20 (green) milliseconds at unloaded shortening. The single exponential fitting with the experimentally observed time scales well match the simulated tensions (τ_*R*_ = 34 ms for the rise after activation, τ_10_ = 28 ms after 10% of muscle length shortening, and τ_5_ = 24 ms after 5% of muscle length shortening). Also the time delay are quite similar to the experimental data (Linari et al., 2015) (18, 8, and 1 ms for the activation, 40 and 20 ms protocols respectively. Experimental data are 21, 2, and 0 ms).

Parameters are defined following some assumptions required to reproduce the contractile response of frog muscle fibers at low temperature. Some assumptions are shared between the two models: the time constant of the rising phase after activation must be comparable to the one observed experimentally in Linari et al. (2015) (τ_*R*_ = 34 ms); about one third of myosin motors are attached during isometric tetanus plateau (Piazzesi et al., 2007); unloaded velocity of shortening must be comparable to experimental value. Moreover, in the MS model we also require that in the relaxed muscle about 5% of myosin motors are in the ON state, as hypothesized in Linari et al. (2015), and that the mechanosensing recruitment reaches the saturation at *T* greater than 0.5 *T*_0_. The tension-velocity curve simulated by the conventional model (Figure 3A, red squares) shares the common limit described in the introduction: despite both the maximum velocity and the rising phase after activation (Figure 3B, blue line) are comparable with the experimental data, the velocities at intermediate forces are sensibly lower than the experimental data obtained on the same muscle type (Piazzesi et al., 2007), (*Rana temporaria*, filled diamonds in figure). This reduces the power output in this range of forces to unrealistic low values.

The limits of the conventional approach are clearly detectable when the tension is allowed to recover isometrically after a period of unloaded shortening (Figure 3B). The time constants, for the 20 ms (green line) and the 40 ms (red line) drop in tension, are equal to each other, as expected by a model without a feedback from the tension. With the current parameters, the simulated time constant is comparable to the one observed for the 20 ms drop but it is pretty higher than that of the 40 ms drop. A different choice of parameter would modify the outcome, but the two rates can be made different only introducing further hypotheses in the conventional model.

The MS model includes the stress dependent transition between the two detached states. It is able to fit excellently the tension-velocity curve (Figure 3A, blue dots), while keeping the proper fitting for the rising phase during isometric activation (Figure 3C, blue curve). Consequently, the power output at intermediate level of velocity of shortening is preserved at the physiological values. The model is able to keep a high maximum power thanks to a relatively high attachment and detachment rates, while the rising phase is slowed by the ON-OFF transition. These results strongly support the idea that the mechanosensing mechanism in the recruitment of ON or active myosins may solve the long standing conflict described before.

**Figure 4:**
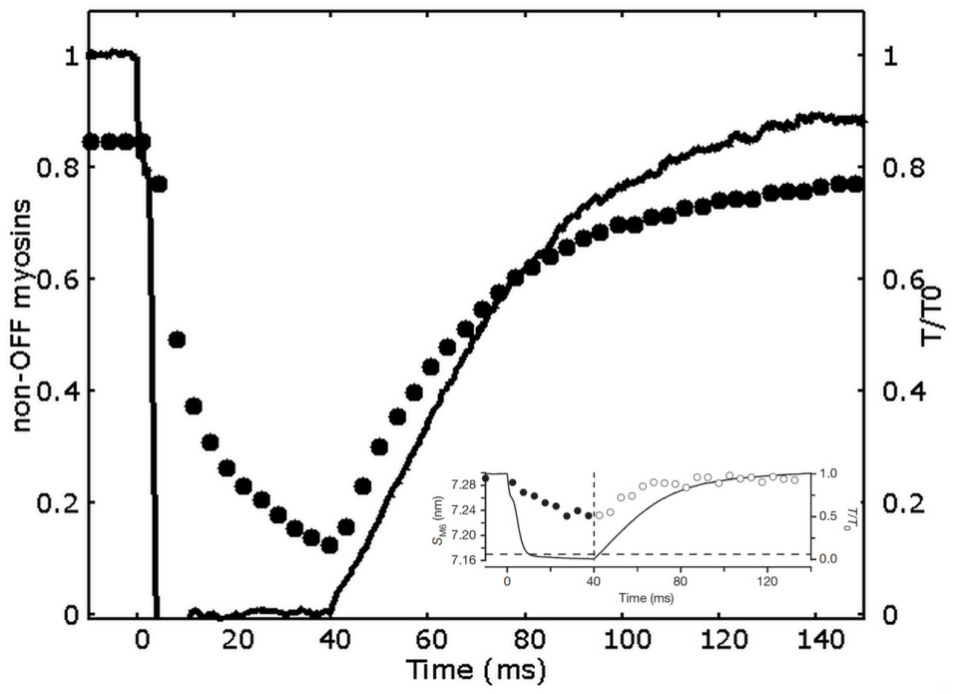
**Decrease in the non-OFF myosin during a 40 ms drop in tension**. Relative tension (continuous line) and relative number of non-OFF state myosins (filled circles) vs. time. Non-OFF state myosins are given by the sum of ON and attached myosin motors. The behavior semi-quantitatively follows the SM6 signal (data in the inset, adapted by permission from Macmillan Publishers Ltd: Nature Linari et al., 2015, copyright 2016).

The simulated tension transients after unloaded shortening are shown in Figure 3C. The curves for the tension development after initial activation, and after an imposed shortening at zero tension for 20 and 40 ms, closely follow the single exponential fitting with time constants respectively of 34, 24, and 28 ms, and shifted by a proper time delay as done in Linari et al. (2015). These values are the same as the ones observed experimentally, and also the time delays are quite similar (see caption of Figure 3). Interestingly, the characteristic timing of the observed mechanosensing mechanism, is compatible with the attachment and detachment rate constants required to fit the high power output at intermediate velocities. Differently to the conventional model, now we can reproduce a lower time constant after longer zero-tension periods, because a higher number of myosin motors move to the OFF state in MS model, thanks to the mechanosensing system. The ON-OFF transition can be satisfactorily simulated as model mimics the increase of the myosins in OFF state during ramp shortening, roughly following what observed experimentally (Figure 4).

**Figure 5:**
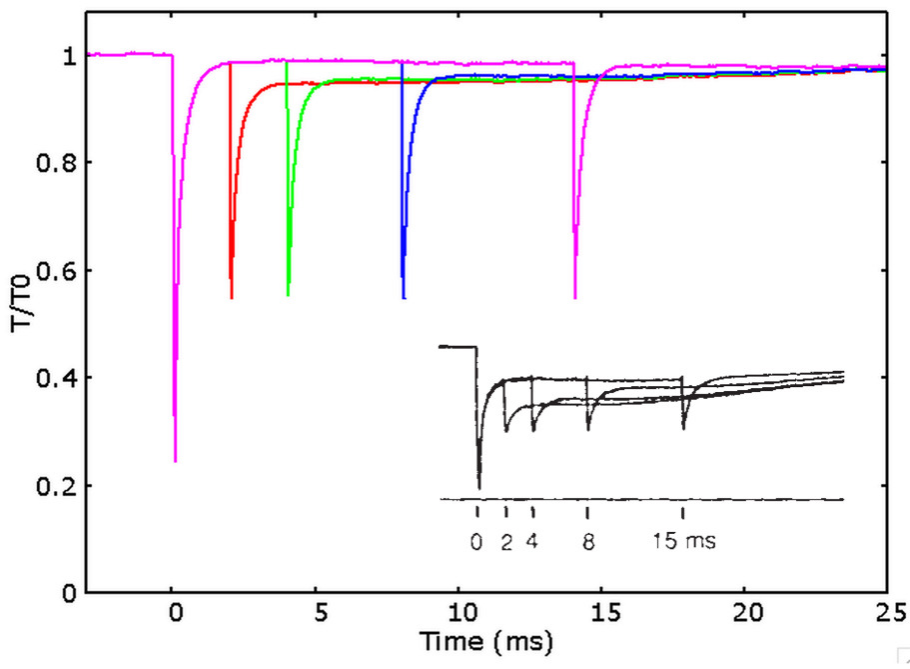
**Fast recovery of the power stroke**. Simulated relative tension vs. time after a conditioning length step of 5 nm and a test step of 2 nm applied after 2 ms (red), 4 ms (green), 8 ms (blue), and 14 ms (pink) after the conditioning step. The tension recovered after the test step increases with the time delayed after the conditioning step, semi-quantitatively fitting the experimental data (shown in the figure, adapted by permission from Macmillan Publishers Ltd: Nature Lombardi et al., 1992, copyright 1992).

It is important also to test the predictive ability of the model in relation to another behavior which has already been connected to the above discussed conflict, the so-called fast recovery of the power stroke. This behavior has been shown first about 25 years ago by Lombardi et al. (1992). The tension recovery after a small “test” step in length, which follows a larger “conditioning” step during steady state of the isometric contraction, increases with the time interval between the two steps, showing a greater recovery in tension already after 4 ms. The recovery increases more and more up to 15 ms after the first step. This evidence is incompatible with the slower time scale of the attachment detachment process, as inferred during the rising phase in conventional models. In Figure 5 are shown the simulations of the experimental protocol used for the multiple steps in Lombardi et al. (1992). The model is able to almost quantitatively fit the experimental behavior, clearly showing an improved capacity to recover the original tension with the delay of few milliseconds from the conditioning step.

**Figure 6:**
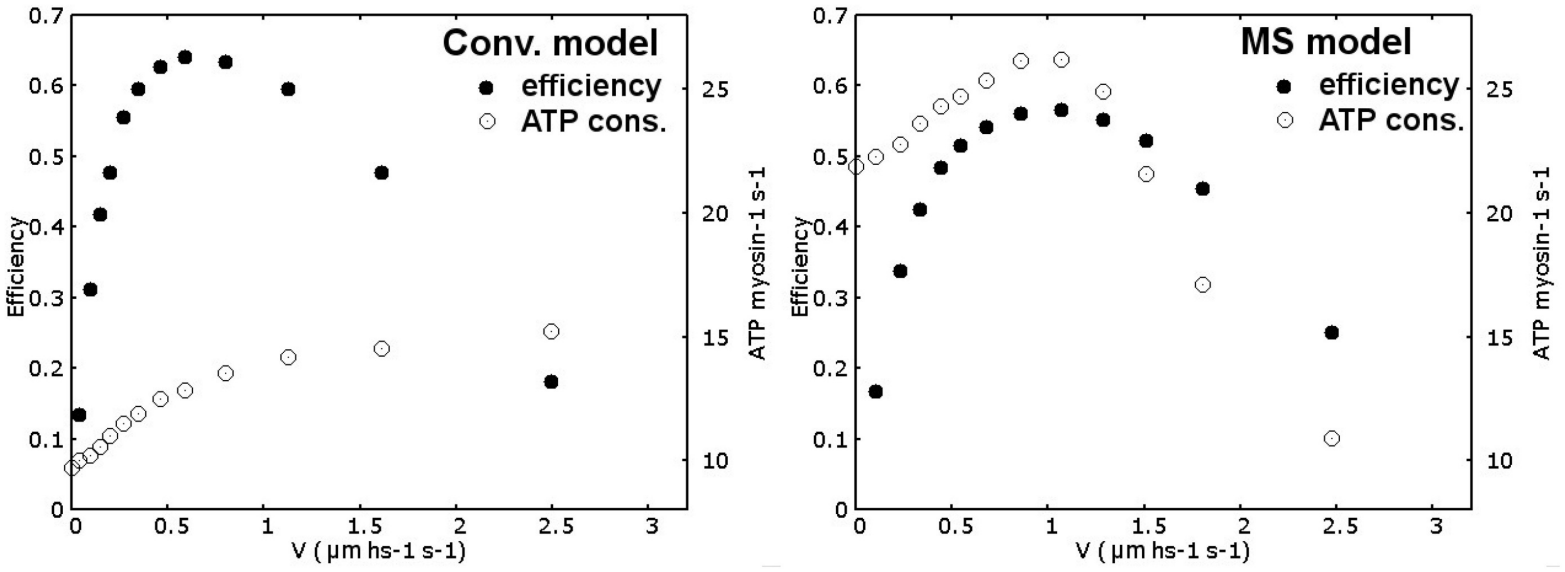
**Efficiency and ATP consumption**. Efficiency (filled dots) at different shortening velocities simulated by the conventional model (left panel) and MS model (right panel) is computed as work done (force times displacement) divided by ATP consumed (85 pNnm per ATP) in a given amount of time. ATP consumption per myosin per second (empty dots) is also reported for both models. While in the conventional model it is monotonically increasing, MS model predicts a maximum in energy consumption at intermediate velocities.

Even though the model has been defined primarily to analyze the force-velocity relationship and the rise in tension after activation, the predicted energetics is, to some extent, compatible with experimental observations, and its analysis is of some interest to understand the potential influence of the mechanosensing mechanism in physiological situations. We first introduce some considerations about the efficiency evaluation. Energy consumption is associated to one ATP splitting, which is about $20\;K\bar{T}$, at the detachment of one myosin motor. Detachment from the pre-power stroke state may be prevented in real muscle, or it may be likely possible but without the associated ATP splitting. These situations would increase the predicted efficiency, decreasing the ATP consumption, compared to allowing the detachment from any attached state. Despite that this behavior is not fully understood, so, as the simplest hypothesis, in both the conventional and the MS models, we imposed that the motors can detach from any attached state, also in the pre-power stroke, consuming one ATP molecule. Moreover, *F*_*atp*_ component in Equation (**1**) represents the ATP bias in acto-myosin conformational changes, but also the H57 hypothesis introduce a bias in the attachment, generating tension in a preferred direction. This mechanism also requires energy consumption. To account for this component, we used only a fraction of the total ATP energy in the definition of *F*_*atp*_ (in particular $8\;K\bar{T}$ for each minimum), but the energy consumption associated to the H57 hypothesis is difficult to define and its value affects the predicted efficiency. Anyway, computing the total work as the force multiplied the displacement in a given amount of time and dividing by the energy consumed, associating one ATP splitting with $20\;K\bar{T}$, for each detachment event, the simulated maximum efficiency (filled dots in Figure 6) results compatible with the values reported in literature for frog muscles (between 0.45 and 0.56 Smith et al., 2005), for both models. Notably, the maximum efficiency in the MS model is attained at about 1/3 of the maximum velocity while in the conventional model it is located around 1/5 of the this value, which is more similar to experimental data. The ATP consumption during isometric contraction (empty dots in Figure 6) is higher for the MS model (21.87 ATP/myosin/sec.) than the conventional model consumption (9.72 ATP/myosin/sec), as a consequence of the higher attachment and detachment rates of the myosin motors used to increase the power output at intermediate velocities. Both values are higher than experimental data (He et al., 1999), but while the conventional model reaches physiological values since very small shortening velocities, in the MS model it is sensibly higher until intermediate velocities. This limit may be solved considering a steric effect in the attachment probability. The attachment may be limited during the isometric contraction by the lower availability of free actin sites, the availability then increases in isotonic contraction. This explanation would not work without the mechanosensing mechanism because it would predict anyway a faster rise in tension after activation. The steric effect would also increase the efficiency at slow velocities of shortening, shifting the maximum in MS model toward more physiological values, partially explaining the above mentioned discrepancy. Another interesting observation in the predicted energetics, is the non-monotonic behavior of energy consumption associated to the mechanosensing mechanism. At low velocities of shortening, the muscle consumes more ATP per unit of time than the isometric contraction, but at higher velocities, when the tension sustained by thick filament decreases below 0.5*T*_0_, the number of available myosin motors decreases and the rate of ATP consumption drop. Experimentally, enthalpy production rate has been shown to increase with contraction velocity al high and intermediate loads. At higher velocities, this dependence is less pronounced and it may even decrease (Hill, 1964; Kushmerick and Davies, 1969; Kushmerick et al., 1969; Stainsby and Barclay, 1976) and see also (Barclay et al., 2010). In particular, in Homsher et al. (1981) it has been clearly stated that the observed ATP turnover rate in rapid shortening is not several times higher than the rate observed in isometric contraction, as instead predicted by other conventional models. Interestingly, in that work it has also been hypothesized that the so-called unexplained enthalpy, an extra energy liberated during rapid shortening which cannot be explained by the simultaneously measured chemical reactions, implied a more complicated hydrolisis mechanism than that proposed in the classical Lymn and Taylor cycle (Lymn and Taylor, 1971). They shown how this effect could be explained introducing two states for the cross-bridges, differently populated in isometric contraction and in rapid shortening. Our result support this idea, since the quantitative prediction of the model has been proved to be compatible with a decrease in ATP consumption at high velocity of shortening. Anyway, we did not push further the energetics analysis at this stage, because the model itself is still limited in some aspects. For instance, the two steps of the power stroke are assumed equal for sake of simplicity despite they are probably different (Capitanio et al., 2006). Moreover the model does not include filament compliance. A compliance in one or both the filaments, would introduce a tension dependent displacement of the myosin anchor site on the thick filament relatively to the binding site on the thin filament, affecting the attachment and the detachment probability, especially in the H57 hypothesis used in this model. In fact, it has been shown that the filament compliance influences the apparent attachment and detachment rates of myosin motors, affecting both the tension rise and the tension-velocity curve (Mijailovich et al., 1996; Daniel et al., 1998). The relative importance of the rigid filament hypothesis has to be analyzed but it is over the scope of the present work.

## Discussion

In this study we have shown that the tension dependent recruitment of active motors from the OFF state makes the tension development after activation less sensitive to the acto-myosin cycle rate constants and thus allows higher rate constant of attachment. This mechanism, which is substantiated by recent experimental findings (Linari et al., 2015), solves the long standing discrepancy between the high power output and the slow rise in tension in skeletal muscle modeling. This conflict, surviving since the first model based on molecular mechanism proposed by Huxley (1957), has been previously solved only introducing *ad-hoc* hypotheses on the actin or myosin properties. We have also shown that the experimentally observed kinetics of this mechanism are quantitatively in agreement with our hypothesis. Perturbing in some way ON-OFF kinetics would allow to experimentally test this hypothesis. For instance, increasing, chemically or mechanically, the number of active motors in the relaxed muscle, would generate a faster tension rise after activation without affecting the tension-velocity relationship. Alternatively, perturbing the tension in the thick filament during activation would modify the number of active motors, and consequently affect the mechanical response of the muscle.

The ON-OFF regulation, requires a tension feedback which is likely to be generated by a molecular component other than myosin or actin, like titin or the C-protein. How this feedback information is given to each myosin motor has to be explored experimentally. The tension may modify the state of the non-myosin proteins in the thick filament, affecting the rates of each motor in the same way. Or the extension of the thick filament may disturb the myosin-myosin interaction in the OFF state, inducing a nearest-neighbor cooperative interaction. The latter is required to describe the steep slope in the pCa-force curve (Rice et al., 2003) and the mechanosensing mechanism may then contribute to the known coupling through the tropomyosin filament. Further experimental evidence are required for an explanation of the tension dependence, which is imposed on phenomenological basis in this paper. Nevertheless, in this work we have shown that including a new, experimentally well supported, structural property of the thick filament in muscle modeling is as important as the precise characterization of actomyosin interaction.

## Author contributions

LM contributed to the conception and design of the model, the numerical simulations, and analysis of results, LM and CR contributed to interpretation of results, drafting and critical revision of the article.

## Funding

LM work was supported by European Commission, Seventh Framework Programme (FP7/2007-2013) under Grant Agreement n600376.

### Conflict of interest statement

The authors declare that the research was conducted in the absence of any commercial or financial relationships that could be construed as a potential conflict of interest.
