# Supplementary material for: Mechanosensing in Myosin Filament Solves a 60 Years Old Conflict in Skeletal Muscle Modeling between High Power Output and Slow Rise in Tension
Source: Front Physiol. 2016 Sep 23;7:427. doi: 10.3389/fphys.2016.00427 (PMC5034546; doi:10.3389/fphys.2016.00427)
Supplement: Supplementary file 1 [file DataSheet1.pdf]

1 SUPPLEMENTARY TEXT

A Monte-Carlo approach is used in both models, where the state and position of each myosin is followed at each time step $\Delta t = 10^{-6} s$. At each time step, the algorithm updates the position x_i of the i-th myosin with respect to its anchor on the thick filament, considering the current position of the actin filament, the coupling factor for the myosin motors. In this way, the total force generated is given by:

$$F_{Tot} = \sum_{j=1}^{N_{fil}} \sum_{i=1}^{N_{XB}} k x_{ij} \tag{S1}$$

where x_{ij} refers to the i-th myosin motor in the j-th filament, N_{XB} is the total number of myosin heads, and N_{fil} is the number of filaments in the sarcomere, while k is the stiffness of the myosin motor.
The position of the myosin head is first updated based on the position of the actin filament, z, in the previous time step:

$$x_i(t) = z(t) - z_i^a + x_i^a + n_{ps} d \tag{S2}$$

Here z_i^a and x_i^a are the positions of the actin filament and the myosin pre-stretch, respectively, at the time of attachment of the $i - th$ myosin head. n_{ps} equals 0, 1 and 2 when the myosin head resides in the S0, S1 and S2 minimum, respectively. Having updated the position of each myosin head, the algorithm updates the position of the actin filament by the NewtonRaphson method. In each iteration, the total force is computed by Equation S1.
In both models, rate constants in the attached state are computed through a Kramers-Smoluchovsky approximation of the corresponding Langevin equation of a diffusing, over-damped particle with drag coefficient η in the sinusoidal energy landscape $E_c(x) + 1/2k(x - x_0)^2$ (see Matherials and Methods):

$$\eta \dot{x}_i = -\omega_i(t) E'(x_i) - k(x_i - x_0^i) + \sqrt{\eta \kappa_b \bar{T}} \Gamma(t). \tag{S3}$$

ω is zero when the myosin head is detached and one when it is attached. $\Gamma(t)$ is a random term satisfying $< \Gamma(t) >= 0$ and $< \Gamma(t_1), \Gamma(t_2) >= 2\delta(t_1 - t_2)$ (white noise).

The algorithm for the model requires a pre-step outside the time loop::

0. Generate a matrix of rate constants for each stretching level between -80 nm and 20 nm through a numerical integration of the Kramers-Smoluchivsky approximation of the Langevin equation describing the motion of a particle in the defined potential energy.

Then:

1. Compute the tension acting on each thin filament and computing the new value of k_{01}
2. Define the new stable state of each myosin (OFF, ON, Pre or post power stroke) using the rate constants for the transitions k_{01}, k_{10}, k_{12}, k_{21},k_f and k_b.
3. Update the positions of each myosin head and the actin filament, depending on the setup and external conditions, and calculate the total force.
4. Update the position of the actin filament through an implicit method
5. Increment the time by one time step and return to Step 1 until total time is less than the prescribed

Table S1. Parameter values and their descriptions

Parameter	Value	Description
k	2 pN/nm	myosin stiffness
η	70 $pNns/nm$	myosin drag coefficient
LB	50 nm	half-bare zone
LM	825 nm	half-myosin filament length
LA	1224 nm	actin filament length
Δt	1 μs	time step
$\bar{T}$	$277.15K$	Temperature
K	0.0138 $pNnm/K$	Boltzmann constant
k_{01} MS	$596.8 * ON_f s^{-1}$	OFF-ON rate
k_{10} MS	206.5 s^{-1}	ON-OFF rate
k_{12} MS	92.1 $s^{-1}nm^{-1}$	ON to attached rate
k_{21}^{+} MS	19.1 $s^{-1}nm^{-1}$	attached to ON rate when $x > 0$
k_{21}^{-} MS	490 s^{-1}	attached to ON rate when $x < 0$
k_{01}^{0} conv	$17.0s^{-1}$	OFF-ON rate
k_{10}^{0} conv	7500 s^{-1}	ON-OFF rate
γ conv	40	cooperativity factor
k_{12} conv	30.0 $s^{-1}nm^{-1}$	ON to attached rate
k_{20}^{+} conv	6.2 $s^{-1}nm^{-1}$	attached to ON rate when $x > 0$
k_{20}^{-} conv	500 s^{-1}	attached to ON rate when $x < 0$
m	0.01	minimum ON_f
t_f	200 pN^2	inflection factor for ON_f
d	4.6 nm	first and second power stroke step
F_{atp}	8 $K\bar{T}/d\ pN$	ATP bias of the energy
H	5.7 $K\bar{T}\ pNnm$	Barrier between minima
